# Sensitivity of the Green Alga *Pediastrum duplex* Meyen to Allelochemicals Is Strain-Specific and Not Related to Co-Occurrence with Allelopathic Macrophytes

**DOI:** 10.1371/journal.pone.0078463

**Published:** 2013-10-22

**Authors:** Falk Eigemann, Pieter Vanormelingen, Sabine Hilt

**Affiliations:** 1 Department of Ecosystem Research, Leibniz-Institute of Freshwater Ecology and Inland Fisheries, Berlin, Germany; 2 Institute of Biology, Freie Universität Berlin, Berlin, Germany; 3 Laboratory Protistology & Aquatic Ecology, Gent University, Gent, Belgium; University of Connecticut, United States of America

## Abstract

Interspecific differences in the response of microalgae to stress have numerous ecological implications. However, little is known of intraspecific sensitivities and the potential role of local genetic adaptation of populations. We compared the allelochemical sensitivity of 23 *Pediastrum duplex* Meyen strains, a common component of the freshwater phytoplankton. In order to test for local genetic adaptation, strains were isolated from water bodies with and without the allelopathically-active submerged macrophyte *Myriophyllum*. Strains were assigned to *P. duplex* on the basis of cell shape and colony morphology and only *P. duplex* strains that belonged to the same lineage in an ITS rDNA phylogeny were used. Inhibition of strain growth rates and maximum quantum yields of photosystem II were measured after exposure to tannic acid (TA) and co-culture with *Myriophyllum spicatum*. Growth rate inhibition varied over one order of magnitude between the *P. duplex* strains. There was no correlation between the presence of *Myriophyllum* in the source location and the sensitivity of the strains to TA or the presence of *Myriophyllum*, suggesting that at least strong unidirectional local adaptation to *Myriophyllum* had not taken place in the studied water bodies. The maximum quantum yield of photosystem II of TA exposed algae decreased, whereas the yield of algae exposed to *M. spicatum* was slightly higher than that of the controls. The ranking of *P. duplex* strain sensitivities differed between the types of exposure (single additions of TA versus co-existence with *M. spicatum*) and the parameter measured (growth rate versus maximum quantum yield), emphasizing the importance of measuring multiple traits when analysing strain-specific sensitivities towards allelochemicals. The observation that sensitivities to allelochemicals vary widely among strains of a single freshwater algal species should be taken into account if evaluating ecological consequences of allelopathic interactions.

## Introduction

Natural populations of phytoplankton show high genetic diversity in ecologically important traits [1-5]. The majority of phytoplankton studies focus on interspecific-sensitivities to toxicants [1,2,6,7], which can span several orders of magnitude [8]. These differences are caused by genetic variability, environmental variability or an interaction between the two [9] and even in single clonal cultures genetic variability may arise rapidly through *de novo* mutations e.g., [10,11]. Often differential natural selection leads to local genetic adaptation of populations to their ambient environment [1,12-16]. Consequently, the strain origin may form the basis of strain-specific responses. For example, Japanese and Australian strains of *Chattonella marina* have different tolerances to high light intensities, correlating with the water clarity of their origin [17]. Similarly, neritic diatom strains were found to be less sensitive to polychlorinated biphenyls (PCBs) than oceanic strains of the same species [1]. In the latter case, it was proposed that an adaptation occurred, as coastal waters are polluted with PCBs. Because coastal waters offer less stable conditions, it was further suggested that neritic strains should be more stress resistant in general [1,2]. However, adaptations specific to a stressor and overall tolerance may or may not occur simultaneously [2]. 

One of the potentially important ecological traits of phytoplankton is their sensitivity towards allelochemicals. Numerous cyanobacteria, algae and submerged macrophytes are capable of producing and releasing allelopathically active compounds that may inhibit the growth of co-occurring phytoplankton species, e.g., [18,19]. Thereby, polyphenolic allelochemical concentrations of 2-4 mg L^-1^ were calculated to occur in macrophyte stands [18]. Recent studies also revealed that epiphytes are susceptible to chemicals released by macroalgae [20], but epiphytic algae and cyanobacteria species were found to be less vulnerable to macrophyte allelochemicals compared to planktonic species [21], potentially due to resistance by co-evolution [22]. Due to different sensitivities, allelochemicals may thus also influence community compositions in the impacted environment [23]. Environmental adaptation and co-evolution were previously suggested to decrease the *in situ* relevance of allelopathic interactions and doubts were raised that allelopathy would even occur between plants that have co-evolved [24]. Based on these findings, the novel-weapon hypothesis was created [25,26], which proposes that some invasive plants may perform better in invaded regions because they introduce unique, species-specific biochemical impacts to native plant and soil microbial communities. The first indications for adaptation of algal populations to allelochemicals were provided by [27], who showed a higher sensitivity of a green algal (*Scenedesmus obliquus*) strain to extracts of the allelopathically active macrophyte *Stratiotes aloides*, when they were isolated from macrophyte-free ponds as compared to a strain from a pond with *S. aloides*. Before, differences in the response of phytoplankton to allelochemicals have mainly been discussed at the species level, e.g., [18,19]. However, support for phytoplankton strain-specific sensitivities to allelochemicals and local adaptation of populations to plant-released allelochemicals requires additional studies comparing a larger number of strains from several origins. 

In the present study, we compared the sensitivities of 23 strains of *Pediastrum duplex* Meyen (a planktonic green alga common in eutrophic freshwaters) to polyphenolic allelochemicals. Algal strains were isolated from two macrophyte-free lakes (13 strains) and two lakes with stands of allelopathically active macrophytes (*Myriophyllum* spp.). Growth rates and maximum quantum yields of photosystem II of the algal strains were measured after single additions of a synthetic polyphenolic allelochemical (tannic acid, TA), and in co-existence with experiments involving *M. spicatum*, which is known for exudation of polyphenols. We hypothesized that (1) *P. duplex* strains exhibit significantly different sensitivities to allelochemicals, and (2) that sensitivities of strains isolated from lakes with *Myriophyllum* spec. are lower than those of strains from macrophyte-free lakes due to local genetic adaptation. 

## Materials and Methods

### Ethics statement

*Myriophyllum spicatum* was harvested from Lake Flakensee with permission of the Brandenburg ministry of environment, health and consumer protection. Phytoplankton samples from Lake Müggelsee, Lake Krumme Laake and Lake Teufelssee were taken with permission of the Berlin Senate, department for urban development and environment. Phytoplankton samples from Lake Molenmeers and Kalken were taken with permission of the Belgium NGO Natuurpunt.

### Test organisms and culture conditions

Live phytoplankton samples were collected from 4 different ponds or lakes (Table 1), either containing no macrophytes or dense stands of submerged *Myriophyllum spicatum* (pond “Molenmeers”) or *M. verticillatum* (lake “Krumme Laake”). Both *Myriophyllum* species are known to produce and exude water soluble polyphenolic allelochemicals affecting several phytoplankton species [18,28,29]. In Krumme Laake (KL), *M. verticillatum* stands were restricted to one bay, so that additional water samples could be obtained from a macrophyte-free bay (300 m distant to macrophyte stands) to test for intra-lake differences in strain sensitivities. *P. duplex* strains, recognized based on the diagnostic cell shape and presence of intercellular spaces [30], were isolated from water samples by micropipetting [31]. Cultures were first grown in WC (Wright`s Chu #10) medium [32] (without pH adjustment or vitamin addition) in well plates at 18 ± 0.5°C and 20-30 µmol photons m^-2^ s^-1^. For experiments, they were grown in modified [33] MIII (ionic-composition similar to the water of the highly eutrophic Lake Müggelsee) medium [34] at pH 7.9 ± 0.1, 20 ± 0.5°C and 80 µmol photons m^-2^ s^-1^ under 12:12 h light:dark conditions in a conditioning cabinet. Cultures were shaken gently at 60 r.p.m. Strains of some additional *Pediastrum sensu lato* species for phylogenetic comparison were isolated and cultured from the same four ponds as well as from Lake Müggelsee (MUGGEL5, 8, 9, 10, 13, 14, coordinates 52°50´68.44N, 13°42`47.32”E, sampled 15/07/2010).

**Table 1:** Characteristics of the water bodies used for isolation of *P. duplex* strains.

Water body	Molenmeers (Molen)	Krumme Laake (KL)	Teufelssee (Teufel)	Kalkengracht (Kalken)
Coordinates	51°01’57.25” N	52°43´48.78“N	52°41`81.44”N	51°01’38.91”N
	3°55’07.61”E	13°63´50.56”E	13°68`87.72”E	3°55’19.05”E
Sampling date	8 July 2010	20 July 2010	20 July 2010	8 July 2010
Isolation date	9 July 2010	24-30 July 2010	24-30 July 2010	9 July 2010
Area (ha)	0.14	3	1.2	3.1
Mean depth (m)	1.5	4	2	2
Presence of macrophytes	*M. spicatum*	*M. verticillatum* Macrophyte-free bay	None	None	
*P. duplex* strain numbers	13, 14, 15, 20, 22	3, 5, 8, 9, 14 N2, N10	4, 6, 8	49, 50, 55, 56, 57, 58, 59, 60		

### Phylogenetic analysis

Given the high incidence and diversity of algal (pseudo)cryptic species, including *Pediastrum duplex* [35], ITS rDNA sequences were used for assigning *P. duplex* strains to species level. *P. duplex* strains were randomly chosen from within each pond for sequencing, until a total of 50 sequenced *P. duplex* strains were reached. For phylogenetic comparison, the ITS rDNA of two *P. duplex* var. *elegans*, two *P. boryanum*, five *P. tetras*, and four *P. angulosum* strains were also sequenced. *Pediastrum* was recently split in five genera [36]; however at least part of this revision is not well-supported [35], thus we continue to apply the previous wide genus concept. The DNA sampling and extraction methods, polymerase chain reaction, as well as sequencing were performed as described by 31, with the first exception that purification for the PCR product was achieved enzymatically, using Exonuclease I to remove leftover primers and shrimp alkaline phosphatase to remove dNTPs, and the second exception that DITS2 (5’-CGC TGC GTT CTT CAT CGA TG-3’) and DITS3 (5’-ACA ACT TTC AGC AAT GGA TGT C-3’) were used as sequencing primers [37]. All sequences were submitted to GenBank (accession numbers KF536748-KF536810). Phylogeny reconstruction was done with Bayesian Inference using MrBayes version 3.1.1 [38]. The GTR + G + I model was applied with four rate categories. No initial values were assigned to the model parameters. Two runs of four Markov Chains (one cold and three heated) were run for 10 million generations and sampled every 250 generations. This yielded a posterior probability (PP) distribution of 40,001 trees. After exclusion of 20,000 ‘‘burn-in’’ trees, PPs were calculated by constructing a 50% majority-rule consensus tree. 

### Algal concentrations and growth rate

Growth rates of algae were determined based on chlorophyll (chl) fluorescence measured with a MAXI-Imaging-PAM (exp 1, pulse-amplitude-modulated) or a Phyto-PAM (exp 2) fluorometer (Fa. Walz, Effeltrich, Germany). Before measurement, the cultures were dark adapted for 15 min. For Phyto-PAM measurements, 2 mL of algal suspension were placed in a cuvette equipped with a magnetic bar and a stamp. Measuring frequency was set to 2 and damping to 3. Maxi-Imaging-PAM measurements were conducted directly in 24-well plates (cellstar, Greiner bio-one, Frickenhausen, Germany) with 2 mL of algal culture. Minimal fluorescence F_0_ [39] was determined as proxy for chl *a* content of the algal cultures [40]. As we did not convert data into real chl *a* values, we subsequently use the term chl F_0_. With these data, growth rate µ was calculated as: 

$${\text{μ (d}}^{\text{-1}}{\text{) = ln (ch1 F}}_{\text{0}}{\text{ (t}}_{\text{x}}{\text{) - chl F}}_{\text{0}}{\text{ (t}}_{\text{0}}\text{))/t,}$$

where t is time in days, chl F_0_ (t_0_) is the chl F_0_ value at day 0, and chl F_0_ (t_x_) is the chl F_0_ value at day x.

This calculation is valid for exponentially growing cultures consistently measured at the same time of the day [33], if biomass and light intensity are kept below a critical level [41]. Because changes in the culturing may also affect fluorescence values [42], only strains that were kept under identical growth conditions were compared.

Maximal quantum yields of the photosystem (PS) II (hereon termed photosynthetic yields) were determined after applying a single saturation pulse [39] based on the equation 

$${\text{Y=(F}}_{\text{v}}-{\text{F}}_{\text{m}}{\text{)/F}}_{\text{m}}\text{,}$$

where Y is the maximum quantum yield, F_v_ is the variable fluorescence (difference between F_0_ after dark-adaption and F_m_ after the saturation pulse), and F_m_ the maximal fluorescence after the saturation pulse.

### Growth rate and photosynthetic yield inhibition by tannic acid (exp. 1)

In the first experiment, we compared the sensitivities of 23 *P. duplex* strains to 10 mg L^-1^ of the polyphenolic allelochemical tannic acid (TA), which is comparable to polyphenol concentrations possible under *in situ* conditions in macrophyte stands [18]. TA is a common, commercially available polyphenol consisting of a mixture of gallotannins. It has a high water solubility (2850 g L^-1^, Fluka, filling code: 403955/1 64400), has also been detected in *Myriophyllum* [43] and is closely related to the allelopathically active gallo- and ellagitannins found in *M. spicatum* [18] and *M. verticillatum* [28]. TA was shown to inhibit growth and photosynthesis of phytoplankton species, e.g., [33,44]. To derive at a final TA concentration of 10 mg L^-1^, 1.8 mL of an 11 mg L^-1^ TA stock solution was mixed with 200 µL of algal suspension. This TA concentration does not change the pH value in the buffered medium [44]. The TA concentration was based on pre-experimentation with 0, 1, 5, 10, 20 and 50 mg L^-1^ TA, where 10 mg L^-1^ TA were found to inhibit but not kill the algae and thus suited best for our study. Pre-experiments were carried out like exp. 1 (explained below). Although single additions of allelochemicals are representing *in situ* conditions less good than a continuous release by donors, this approach is most often applied in aquatic allelopathy research as it allows controlled conditions, a better reproducibility and excludes interferences between the plants and the algae [19]. The experiment was conducted in inert, sterile 24-well plates (cellstar, Greiner bio-one, Frickenhausen, Germany) in a conditioning cabinet with light from above (12:12 hour dark:light period with 200 µmol photons m^-2^ s^-1^ ) and at 20 ± 0.5 °C. Algal cultures were kept in the exponential growth phase before subjecting them to experiments to assure comparable physiological states. Each well contained 2 mL of algal culture with or without TA and a starting concentration of chl. F_0_ = 10 µg L^-1^ (Phyto-PAM measurements). The plates were shaken gently at 60 r.p.m. Daily and at the same time of day, chl fluorescence and photosynthetic yields were determined as described above. Experiments were run with four replicates with exponentially growing cultures and lasted for 3 days. Growth rates were calculated from day 0 - 3, and the photosynthetic yields were calculated each day. Several values are missing for days 0 and 3, due to technical problems with the photosynthetic yield measurements.

### Growth rate and photosynthetic yield inhibition in co-existence experiments with *M. spicatum* (exp. 2)

In the second experiment, we compared the sensitivity of *P. duplex* strains to *M. spicatum* in more ecologically relevant co-existence experiments [28,33,45]. *M. spicatum* is known to produce and exude allelochemicals that inhibit phytoplankton, with the hydrolysable polyphenol tellimagrandin II as the major inhibiting compound [18]. Polyphenolic substances may account for up to 30% of the dry weight in the genus *Myriophyllum*, with the highest amount in growing apical tips [28,29]. Erlenmeyer flasks (500 ml) were filled with 450 ml of MIII medium and 3.75 ± 0.25 g (fresh-weight (FW)) apical parts of *M. spicatum* (15 cm long) or plastic plants as controls. The apical parts were harvested 4 days in advance and kept in tab water in order to prevent allelochemical or nutrient leaching from the wounds. *M. spicatum* originated from Lake Flakensee and was maintained in the lab with tab water under artificial light in plastic boxes, rooted in the sediment. At the day of the experiment, apical parts were rinsed carefully with distilled water to remove attached algae. Sterile sausage skin dialyse bags regenerated cellulose Wienie-Pak Skinless Sausage Casings (Devro Teepak, Scarborough, Ontario, Canada), with a molecular cutoff weight of 7000 (ca. 30 cm long), were pulled over 40 ml bottomless Schott-bottles, fixed with rubber clamps and filled with 50 ml of algal culture with a starting concentration of chl. F_0_ = 15 µg L^-1^ (Phyto-PAM measurements). Experimental conditions were the same as algal maintaining conditions. Exp. 2 was carried out with four replicates per treatment and with exponentially growing cultures for both the *Myriophyllum*-treatments and controls. Chl. F_0_ and yields were measured daily, beginning at the same time of the day. The experiment lasted for 3 days and growth rates were calculated from day 0 - 3. Photosynthetic yields were calculated each day.

### Statistical analyses

Because the data were not normally distributed and showed no homogeneity of variance (even after transformation of the raw data), non-parametric Mann-Whitney-U tests (MWU) were used to compare growth rates and photosynthetic yields between treatments and controls for each *P. duplex* strain.

Inhibition levels of the growth rates and the photosynthetic yields were calculated by inhibition (%) = (V_C_ - V_T_)/V_C_ * 100, where V_C_ is the mean value of the controls, and V_T_ is the value obtained from the treatment. Subsequently, the means of the inhibition levels of the treatment replicates were calculated.

Kruskal-Wallis tests were used to test for differences in sensitivity levels between the strains using the %-inhibition values. 

Comparisons of inter-lake strain sensitivities were performed using the %-inhibition values and analyses of variance (ANOVA) with subsequent Tukey post-hoc tests, at the *p* < 0.05 significance level. Impacts of the strain origin on sensitivity were determined by Mann-Whitney-U tests, where % inhibition was pooled amongst all strains from the macrophyte-infested water vs. all strains from the macrophyte-free water. Correlations of sensitivity levels between both experiments and between yields and growth rates in the same experiments were made by Spearman rank correlations using %-inhibition. A correlation between inhibition levels in % and control growth rates was performed by regression analysis. All statistical analyses were completed with the software package PASW 17 (SPSS).

## Results

### Species and strain selection

**Figure 1:**
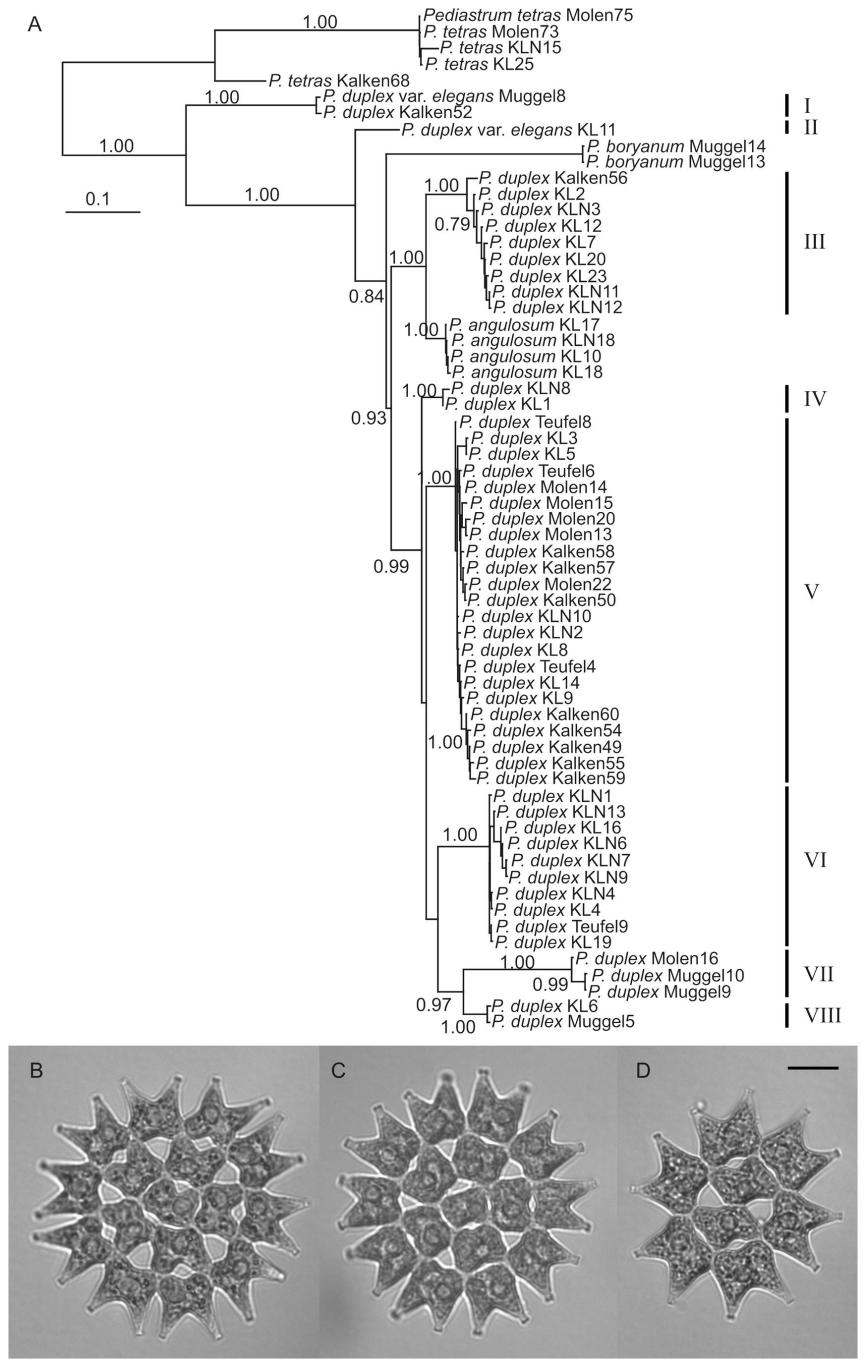
ITS rDNA phylogeny of *Pediastrum* strains (A) and light microscopy photographs of three of the *Pediastrum* duplex strains from lineage V (B-D), used to study sensitivity to polyphenolic allelochemicals. (A) Most likely phylogeny from a Bayesian Inference analysis. Posterior probabilities > 0.70 are shown at the respective nodes. The different *P. duplex* lineages recovered are numbered I-VIII. (B) *Pediastrum* *duplex* strain KL5, (C) Kalken49, (D) Molen20.

The ITS rDNA phylogeny showed that the *Pediastrum duplex* strains belong to no less than 8 lineages, numbered I-VIII, which presumably represent different species (Figure 1A). Based on this phylogeny, the 23 strains from the most abundant *P. duplex* lineage (lineage V in Figure 1A), containing strains from all four study ponds, were selected for subsequent experiments.

### Strain-specific sensitivities to TA (exp. 1)

**Figure 2:**
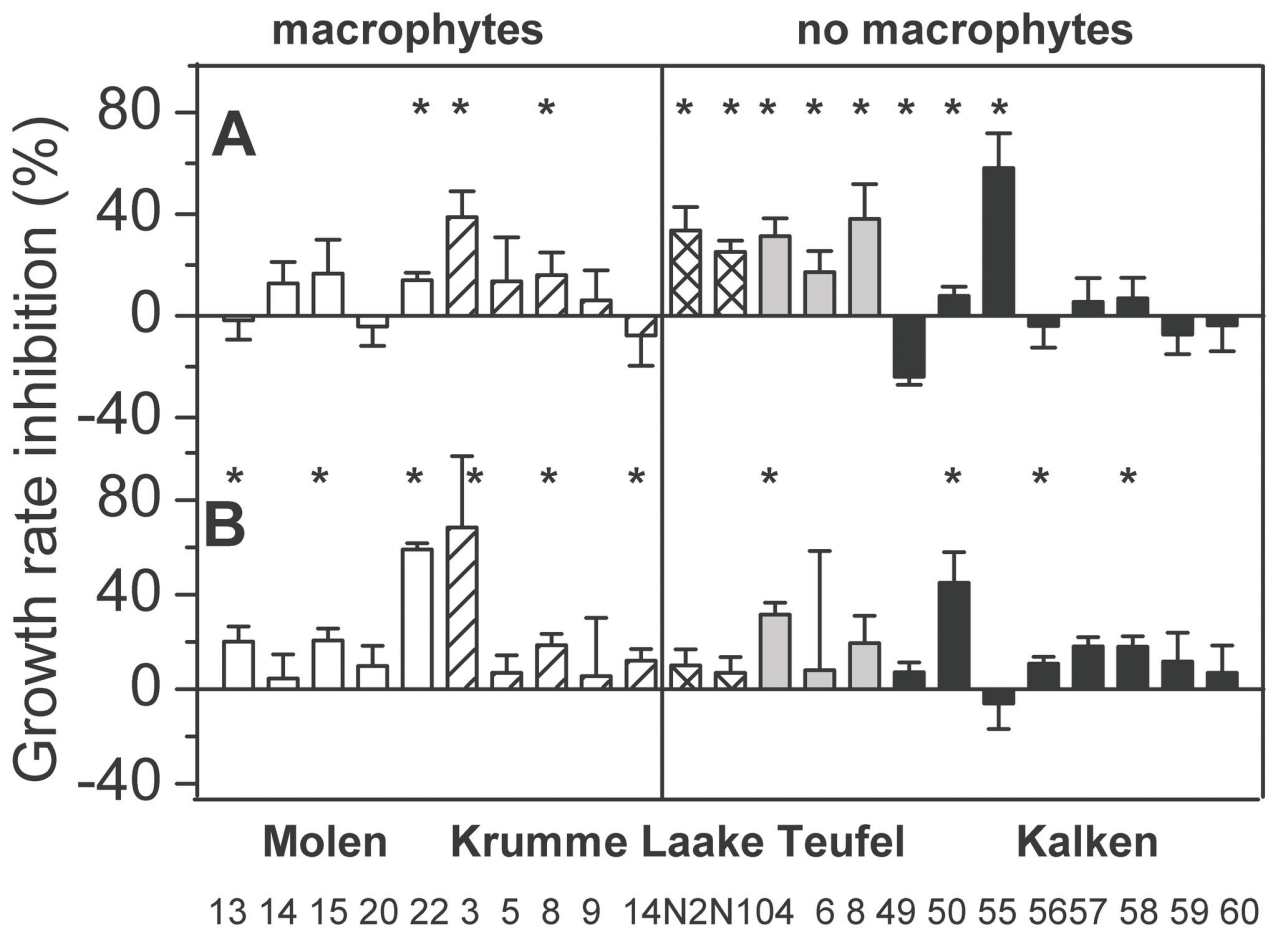
Inhibition of the growth rates of 23 *Pediastrum duplex* strains. Growth rate sensitivity of *Pediastrum* *duplex* isolated from lakes with presence or absence of allelopathically active macrophytes as compared to controls after 3 days of incubation with tannic acid (A) or in co-existence with *Myriophyllum* *spicatum* (B). Asterisks indicate a significant difference between the treatments and controls based on Mann-Whitney U tests. Error bars indicate the standard deviation. The strain numbers are given on the X-axis.

Growth rates of 10 out of the 23 *P. duplex* strains were significantly lower in the presence of TA, while the growth rate of one strain (Kalken49) was significantly increased by TA (Figure 2). Between the strains, significant differences in sensitivities were observed, ranging from 24% (Kalken49) increase to a maximum of 58% (Kalken55) growth rate inhibition (Kruskal Wallis test, *p* < 0.001). Testing the effect of strain origin by comparing sensitivities of strains between the four lakes revealed two different sensitivity levels, with all strains from Kalken (mean inhibition 5%, data not shown) being least sensitive and Teufel (mean inhibition 29%, data not shown) being the most sensitive (one-way ANOVA with subsequent Tukey post-hoc tests, *F* = 6.05, *p* < 0.001, Figure 2A). *Myriophyllum* presence in the pond or lake had no significant effect on strain sensitivities to TA (MWU test between macrophytes vs. no macrophytes, *p* = 0.45). 

**Figure 3:**
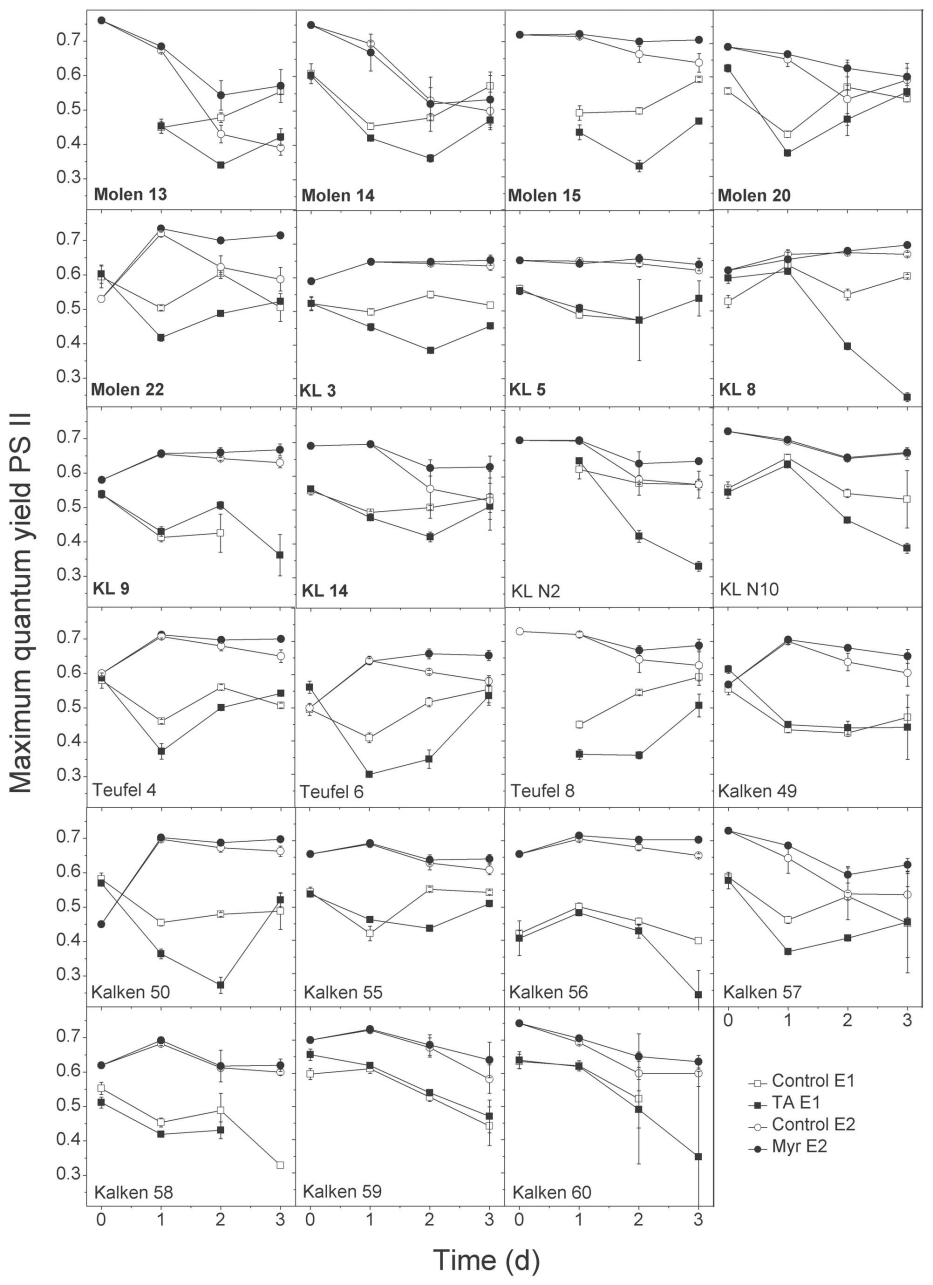
Maximum quantum yields of photosystem II of 23 *Pediastrum* *duplex* strains isolated from lakes with presence or absence of allelopathically active macrophytes during 3 days of exposure to tannic acid (squares) or *Myriophyllum* *spicatum* (circles). Open symbols refer to the controls, and black symbols refer to the allelochemical treatments. Strains written in bold originated from macropyhte-dominated lakes. Error bars indicate the standard deviation.

The photosynthetic yields mostly declined during the first 24 h in the TA treatments (exceptions: KL8, KLN10, Kalken56) and, to a lesser extent, in the controls (exceptions: Molen13, KL8, KLN10, Kalken56, Kalken59; Figure 3). This resulted in significantly inhibited yields by TA addition for 14 strains at day 1 (Figure 3, Table 2) and significant differences in sensitivities (Kruskal Wallis test, *p* < 0.001). By day 2 and/or 3, most of the photosynthetic yields increased (Figure 3). On day 2, 16 strains were significantly inhibited by TA and one strain was significantly enhanced (KL9) by TA (Table 2), showing significant different sensitivities (Kruskal Wallis test, *p* < 0.001). On day 3, the photosynthetic yields of 7 strains were significantly inhibited compared to the controls, and different sensitivities were again observed (Kruskal Wallis test, *p* < 0.001). The inhibition of the photosynthetic yield was not correlated to the presence of *Myriophyllum* at the strain origin (MWU-tests, *p* = 0.52, 0.61, 0.07 for day 1, 2 and 3, respectively). Due to technical problems, several measurements on day 0 and day 3 yielded defective data, which were excluded from the analyses (Figure 3). A correlation between yield inhibition and growth rate inhibition by TA was found for day 3 (Spearman, *r* = 0.27, *p* = 0.02, but not for day 1 and 2 (Spearman, *r* = 0.17, *p* = 0.11 and *r* = -0.08, *p* = 0.45, respectively).

**Table 2:** Results of Mann-Whitney-U tests (*p* values) comparing the maximum quantum yields of PS II between treatments and controls for the respective days and experiments.

Lake		Strain no.	Tannic acid				*Myriophyllum spicatum*			
			Day 0	Day 1	Day 2	Day 3	Day 0	Day 1	Day 2	Day 3
Molen	macrophytes	13	-	0.77	**0.02**	**0.02**	1	**0.03**	**0.02**	**0.02**
		14	0.24	**0.02**	**0.02**	**0.02**	1	0.75	1	0.47
		15	-	**0.02**	**0.02**	**0.02**	1	0.09	**0.02**	**0.02**
		20	**0.02**	**0.02**	**0.02**	**0.02**	1	0.16	**0.04**	1
		22	0.37	**0.02**	**0.02**	0.56	1	0.04	**0.02**	**0.02**
KL		3	0.77	**0.02**	**0.02**	0.16	1	0.87	0.19	0.10
		5	0.15	**0.04**	1	-	1	0.15	0.1	0.21
		8	0.08	0.07	**0.02**	**0.02**	1	0.41	0.19	**0.02**
		9	0.66	0.15	**0.02**	-	1	0.75	0.03	**0.02**
		14	0.39	**0.02**	**0.02**	0.48	1	0.74	0.14	**0.04**
KL	no macrophytes	N2	-	0.08	**0.02**	**0.02**	1	0.62	0.08	**0.02**
		N10	0.25	**0.02**	**0.02**	0.06	1	0.13	0.65	0.74
Teufel		4	0.39	**0.02**	**0.02**	0.06	1	0.44	0.10	**0.02**
		6	**0.03**	**0.02**	**0.02**	0.39	1	0.88	**0.02**	**0.02**
		8	-	**0.03**	**0.02**	**0.02**	1	1	0.18	0.06
Kalken		49	**0.02**	**0.04**	0.25	1	1	0.34	**0.02**	0.06
		50	0.39	**0.02**	**0.02**	0.39	1	0.19	0.09	**0.02**
		55	0.25	**0.02**	**0.02**	**0.02**	1	0.65	0.37	**0.03**
		56	1	**0.03**	0.08	0.18	1	0.06	**0.03**	**0.02**
		57	0.77	**0.02**	**0.02**	0.77	1	**0.04**	0.25	0.06
		58	0.08	**0.02**	0.17	-	1	0.12	0.24	0.10
		59	**0.02**	0.47	0.08	0.56	1	0.65	0.65	0.19
		60	0.77	0.77	0.77	-	1	0.3	**0.02**	0.1

Significant differences between treatments and controls are indicated by bold letters. Dashes indicate that no test was possible due to invalid data obtained from the I-PAM. On day 0 of the *Myriophyllum* experiment, only the stock cultures were measured and the obtained value applied for all samples.

### Strain-specific sensitivities to *Myriophyllum spicatum* (exp. 2)

Out of 23 tested strains, growth rates of 10 strains were significantly inhibited when cultured in co-existence with *M. spicatum* (MWU-tests, Figure 2B). The strains had significant different sensitivities ranging from 6% enhancement (Kalken55) to 68% inhibition (KL3) (Kruskal Wallis test, *p* < 0.001, Figure 2B). However, no differences in intra-lake strain inhibition levels were found (one-way ANOVA with subsequent Tukey post-hoc tests, *F* = 0.67, *p* = 0.57, Figure 2B). The presence of *Myriophyllum* in the ponds of strain origin did not affect strain sensitivity towards *Myriophyllum* (on average 23% and 15% inhibition, respectively, MWU-test, *p* = 0.3, data not shown). Moreover, the inhibition of *P. duplex* growth rates by TA and by *Myriophyllum* were not correlated (Spearman correlation, *r* = 0.17, *p* = 0.1). 

The photosynthetic yields of *P. duplex* strains at day 0 varied, but consistently levelled off to values around 0.7 by day 1, with only slight differences (and only 2 were significant) between the controls and treatments (Figure 3, Table 2). Thereafter, the photosynthetic yields of the controls slightly declined (exceptions KL3, KL8), whereas the treatments with *M. spicatum* either stayed constant or decreased less (Figure 3). The photosynthetic yields of 9 strains were significantly greater by day 2 when in the presence of *M. spicatum* as compared to the controls (Table 2). The strains had significantly different sensitivities (Kruskal Wallis test, *p* < 0.001), with Molen13 being the most (26% higher) enhanced strain. By day 3, the yields of 12 strains were significantly greater in the *Myriophyllum* treatments when compared to the controls, showing significant different inhibition levels (Kruskal Wallis test, *p* < 0.001).

Photosynthetic yields of strains from ponds with macrophytes were not different from macrophyte-free water bodies (MWU tests, *p* = 0.38, 0.23 and 0.57 for day 1, 2 and 3, respectively). Enhancement levels of the yields and growth rate inhibition, due to *Myriophyllum* exposure, were not correlated (Spearman correlation, day 1: *r* = -0.16, *p* = 0.13; day 2: *r* = -0.16, *p* = 0.14; day 3: r = -0.05, p = 0.61). The inhibition/enhancement levels of the photosynthetic yields between both experiments were also not correlated (Spearman correlation, day 1: *r* = -0.13, *p* = 0.24; day 2: *r* = 0.02, *p* = 0.84; day 3: *r* = -0.08, *p* = 0.5). 

## Discussion

Our results confirmed the first hypothesis that *P. duplex* strains exhibit significantly different sensitivities to allelochemicals. The 23 tested *P. duplex* strains were highly variable in their response to single additions of TA as well as to the continuous supply of polyphenolic allelochemicals exuded by *Myriophyllum* plants in coexistence experiments. Contrary to our expectations in the second hypothesis, sensitivities of strains isolated from lakes with *Myriophyllum* spec. were not significantly lower than those of strains from macrophyte-free lakes. We thus could not confirm local adaptation of *P. duplex* to polyphenolic allelochemicals. 

### Genetic analysis of *P. duplex* strains:

We found 8 ITS lineages of the isolated *P. duplex* strains, with ITS lineages belonging to other species interspersed between them. This corroborates the presence of large species-level diversity in *P. duplex* with each lineage probably representing a different species. Not taking such hidden species diversity into account in population-level studies may lead to wrong conclusions about the extent of genetic diversity and micro-evolution (adaptation) within and between populations, and we therefore adopted a molecular approach using a fast-evolving marker to make sure the here-used strains belong to a single species. 

### Strain-specific sensitivities towards allelochemicals

Genetic diversity at the strain level has been underestimated in studies of species-specific phytoplankton responses to allelochemicals, but is important given the potential consequences of genetic diversity of primary producers on other ecosystem functions [5]. Even second-order effects of primary producers genetic diversity on higher trophic levels can be expected, such as those reported for seagrass strains [46].

Our results reveal strong strain-specific differences in the sensitivity of *P. duplex* strains from the same ITS lineage to polyphenolic allelochemicals. Between-strain variability is known for a number of physiological and biochemical characters (e.g., [1,4]) and was now shown to be also the case for sensitivities in allelopathic interactions, an important ecological trait of the phytoplankton. Strain-specific differences were shown before in grazing resistance traits in the green alga *Desmodesmus armatus* [5]. The presence of sufficient intra-population genetic variation allowed local genetic adaptation to the grazing pressure in the respective habitats [5]. Furthermore, the high spatiotemporal heterogeneity of toxin production in natural populations of the cyanobacterium *Microcystis aeruginosa* was explained by the detected high inter-strain variability of this trait [47]. 

Our measured strain-specific sensitivities of *P. duplex* to allelochemicals differed depending on the used observation variable (growth rate versus photosynthetic yield), confirming [44], where sensitivities against TA differed between 5 different observation variables, and [7], where chlorophyta strains exhibited different sensitivities to different metals. These differences might be explained by the fact that chemical stressors and their derivatives tend to affect a large number of processes with different pathways [48]. 

Both single TA additions and continuous release of polyphenolic allelochemicals by *M. spicatum* significantly inhibited the growth rates of about half of the tested strains (Figure 2A, B). Nevertheless, only five strains experienced inhibition by both tested allelochemicals. These differences were not caused by changes in growth rates of strains during experimentation i.e., different physiological states (regressions between growth rates of controls and inhibition of treatments: exp. 1: R^2^ = <0.001, *p* = 0.89, exp. 2: R^2^ = 0.006, *p* = 0.46, data not shown). The specificity of polyphenols does not only depend on functional groups, but also on molecular size, shape and structure [18], which depicts another potential reason for different results between the experiments. However, the ellagitannin tellimagrandin II (C_41_H_30_O_26_), the major inhibiting compound in *M. spicatum* [18] and the mixture of gallotannins in tannic acid (C_76_H_52_O_46_) are of similar shape, size and structure, and also their degradation products have anti-algal activities, and thus effects should be qualitatively comparable. A potential reason for sensitivity differences between the experiments are differences in the concentrations of the tested allelochemicals and/or the method of addition. Differences in the response of organisms to different methods of allelochemical addition were previously emphasized [22]. Furthermore, different concentrations of atrazine (a herbizide inhibiting the electron transport from Q_A_ to Q_B_ similar to TA, [49]) caused different rankings of sensitivities of various *S. subspicatus* strains [4], and different TA concentrations resulted in different sensitivity rankings of three algal species [44]. For dense *M. spicatum* stands a release of 2-4 mg L^-1^ polyphenols within 10 days were calculated [18], neglecting microbial and photolytic decomposition. Due to the shorter exposure time in exp. 2 (three days), the allelochemical concentration might be approximately one order of magnitude lower in exp. 2 compared to exp. 1. Thus different polyphenol concentrations may be one factor for the observed differences in sensitivity. Another potential mechanism explaining different sensitivity rankings in both experiments might be the light availability (80 and 200 µmol photons m^-2^ s^-1^ in exp. 1 and 2, respectively). Under low light conditions, the effect of the macrophyte *S. aloides* on the green alga *S. obliquus* was more pronounced than under high light conditions [50]. In our study, however, the inhibition levels were the same between the experiments (t-test, *p* = 0.08). 

Interestingly, the photosynthetic yields of PS II in numerous strains were significantly inhibited by TA, whereas a slight increase was observed in some strains in co-existence with *M. spicatum*. This result was contrary to our expectations because inhibition of PS II activity has been shown for several phytoplankton species if in co-existence with *M. spicatum* [33]. Importantly, lowered light intensities are known to increase photosynthetic yields [51,52]. We used plastic plants in the controls to simulate shading effects by the macrophytes, but cannot fully exclude differences in light qualities and/or quantities between treatments and controls. Another explanation for increased yields in the *M. spicatum* treatments in exp. 2 are measurements of F_m_ that are lower than the actual F_m_ due to state transitions in the PS II [53], a phenomenon known for numerous cyanobacteria and a few green algal species [54,55]. We tested whether state transitions occur for *P. duplex* by measuring PS II quantum yields in 3 strains (Molen14, KL14, Kalken60) with and without dark adaptation. Lowered F_m_ values after dark adaptation were not detected (data not shown), thus, state transitions in *P. duplex* seem unlikely to explain increased yields in the *Myriophyllum* treatments. Increased maximum PS II yields for algae after exposure to humic substances were also observed [56] and explained by interference with the electron transport chain. Further, increased maximum quantum yields were found for the green alga *Chlamydomonas acidophila* by co-limitation for carbon dioxide and inorganic phosphorus [55]. A conclusive explanation for this phenomenon, however, was not provided in any of these studies.

### Correlation of sensitivities with the strain origin (local adaptation)

Adaptations to local conditions are common in phytoplankton ecology (e.g., [1-3,14,15]), but may not be generalized to every trait [57]. The island-like character of limnic habitats especially offers opportunities for local genetic differentiation and adaptation [58]. Depending on the exposure time to certain environmental conditions, physiological changes, epigenetic adaptations (gene regulation and/or gene expression), or genetically based adaptations can occur for phytoplankton populations [13]. We assumed that the selective pressure of polyphenolic allelochemicals released by *Myriophyllum* spp. on *P. duplex* strains is important under the given environments (water bodies dominated by allelopathically-active macrophytes). Responses within one species were shown to vary as a result to the co-occurrence with allelochemicals in terrestrial environments [23]. Further, local genetic adaptation for grazing resistance traits in natural populations of the green alga *D. armatus* that were exposed to contrasting grazing pressures by zooplankton were detected [5], and a fast genetic adaptation of the green alga *Dictyosphaerium chlorelloides* to moderate acidic and metal rich waters was found [16]. Rapid genetic adaptation of phytoplankton populations may be driven by the large genetic variation often found within populations e.g. [5,59,60] as well as fast growth and large population sizes, which increase the chance that beneficial mutations appear. However, contrary to our hypothesis, differences in sensitivities of *P. duplex* strains to allelochemicals were not correlated with the presence of allelopathically active macrophytes in the lake of their origin, and thus we could not confirm local genetic adaptation and the potential co-evolution between allelochemical donor and acceptors as suggested by [24,27]. We can exclude that the observed lack of adaptation was derived by dispersal of the strains, because all sampled water bodies are closed habitats. However, we isolated strains from different sites (macrophyte-dominated versus macrophyte free bay) in Lake Krumme Laake which may have influenced the outcomes, but also sensitivitiy analysis without strains of Lake Krumme Laake did not reveal adaptation to polyphenolic allelochemicals (T-Test, *p* = 0.38 and *p* = 0.2 for growth rate inhibition levels of strains originating from macrophyte-dominated water bodies versus macrophyte-free water bodies for the TA and co-existence experiment, respectively). Because co-evolution between allelochemical donor and acceptor is only one of many possible factors for the observed divergence in strains, additional physiological, ecological or life history traits arising from the different habitats of the strains might be responsible for the observed patterns. 

## References

[1] Fisher NS, Graham LB, Carpenter EJ (1973) Geographic differences in phytoplankton sensitivity to PCBs. Nature 241: 548-549. doi:10.1038/241548a0. PubMed: 4632783. 4632783

[2] Murphy LS, Belastock RA (1980) The effect of environmental origin on the response of marine diatoms to chemical stress. Limnol Oceanogr 25: 160-165. doi:10.4319/lo.1980.25.1.0160.

[3] Wood AM, Leatham T (1992) The species concept in phytoplankton ecology. J Phycol 28: 723-729. doi:10.1111/j.0022-3646.1992.00723.x.

[4] Behra R, Genoni GP, Joseph AL (1999) Effects of atrazine on growth, photosynthesis, and between-strain-variability in *Scenedesmus* *subspicatus* (Chlorophyceae). Arch Environ Con Tox 37: 36-41. doi:10.1007/s002449900487. 10341040

[5] Vanormelingen P, Vyverman W, De Bock D, Van der Gucht K, De Meester L (2009) Local genetic adaptation to grazing pressure of the green alga *Desmodesmus* *armatus* in a strongly connected pond system. Limnol Oceanogr 54: 503-511. doi:10.4319/lo.2009.54.2.0503.

[6] Menzel DW, Anderson J, Randtke A (1970) Marine phytoplankton vary in their response to chlorinated hydrocarbons. Science 167: 1724-1726. doi:10.1126/science.167.3926.1724. PubMed: 5416533. 5416533

[7] Foster PL (1982) Metal resistances of chlorophyta from rivers polluted with heavy metals. Freshw Biol 12: 41-61. doi:10.1111/j.1365-2427.1982.tb00602.x.

[8] Jensen A, Rystad B, Melsom S (1974) Heavy metal tolerance of marine phytoplankton. I. The tolerance of three algal species to zinc in coastal seawater. J Exp Mar Biol Ecol 15: 145-157. doi:10.1016/0022-0981(74)90040-9.

[9] Hoffman AA, Parsons PA (1997) Extreme environmental change and evolution. Cambridge University Press Cambridge, U.K.

[10] Loxdale HD, Lushai G (2003) Rapid changes in clonal lineages: the death of a “sacred cow”. Biol J Linn Soc 79: 3-16. doi:10.1046/j.1095-8312.2003.00177.x.

[11] López-Rodas V, Flores-Moya A, Maneiro E, Perdigones N, Marva F (2007) Resistance to glyphosate in the cyanobacterium *Microcystis* *aeruginosa* as a result of pre-selective mutations. Ecol Evol 21: 535-547. doi:10.1007/s10682-006-9134-8.

[12] Kawecki TJ, Ebert D (2004) Conceptual issues in local adaptation. Ecol Lett 7: 1225-1241. doi:10.1111/j.1461-0248.2004.00684.x.

[13] Lakeman MB, von Dassow P, Cattolico RA (2009) The strain concept in phytoplankton ecology. Harmful Algae 8: 746-758. doi:10.1016/j.hal.2008.11.011.

[14] Thomas MK, Kremer CT, Klausmeier CA, Litchman E (2012) A global pattern of thermal adaptation in marine phytoplankton. Science 338: 1085-1088. doi:10.1126/science.1224836. PubMed: 23112294. 23112294

[15] Härnström K, Ellegaard M, Andersen TJ, Godhe A (2011) Hundred years of genetic structure in a sediment revived diatom population. Proc Natl Acad Sci USA 108: 4252-4257. doi:10.1073/pnas.1013528108. PubMed: 21282612. 21282612 PMC3054006

[16] López-Rodas V, Sanchez-Fortun S, Flores-Moya A, Costas E (2011) Genetic adaptation and acclimation of phytoplankton along a stress gradient in the extreme waters of the Agrio River-Caviahue Lake (Argentina). J Phycol 47: 1036-1043. doi:10.1111/j.1529-8817.2011.01035.x. 27020184

[17] Marshall JA, Newman S (2002) Differences in photoprotective pigment production between Japanese and Australian strains of *Chattonella* *marina* (Raphidophyceae). J Exp Mar Biol Ecol 272: 13-27. doi:10.1016/S0022-0981(02)00034-5.

[18] Gross EM, Meyer H, Schilling G (1996) Release and ecological impact of algicidal hydrolysable polyphenols in *Myriophyllum* *spicatum* . Phytochemistry 41: 133-138. doi:10.1016/0031-9422(95)00598-6.

[19] Hilt S, Gross EM (2008) Can allelopathically active submerged macrophytes stabilise clear-water states in shallow eutrophic lakes? Bas Appl Ecol 9: 422-432.

[20] Bucolo P, Amsler CD, McClintock JB, Baker BJ (2012) Effects of macroalgal chemical extracts on spore behavior of the antarctic epiphyte *Elachista* *antarctica* Phaeophyceae. J Phycol 48: 1403-1410. doi:10.1111/j.1529-8817.2012.01221.x. 27009991

[21] Hilt S (2006) Allelopathic inhibition of epiphytes by submerged macrophytes. Aquat Bot 85: 252-256. doi:10.1016/j.aquabot.2006.05.004.

[22] Reigosa MJ, Sanchez-Moreiras A, Gonzalez L (1999) Ecophysiological approach in allelopathy. Crit Rev Plant Sci 18: 577-608. doi:10.1016/S0735-2689(99)00392-5.

[23] Jensen CG, Ehlers BK (2010) Genetic variation for sensitivity to a thyme monoterpene in associated plant species. Oecologia 162: 1017-1025. doi:10.1007/s00442-009-1501-z. PubMed: 19921272. 19921272 PMC2841263

[24] Rabotnov TA (1982) Importance of the evolutionary approach to the study of allelopathy. Ekologia 3: 5-8.

[25] Callaway RM, Aschehoug ET (2000) Invasive plants versus their new and old neighbors: a mechanism for exotic invasion. Science 290: 521–523. doi:10.1126/science.290.5491.521. PubMed: 11039934. 11039934

[26] Callaway RM, Ridenour WM (2004) Novel weapons: invasive success and the evolution of increased competitive ability. Front Ecol Environ 2: 436-443. doi:10.1890/1540-9295(2004)002[0436:NWISAT]2.0.CO;2.

[27] Al-Sheri AM (2010) Differential sensitivities of different *Scenedesmus* *obliquus* strains to the allelopathic activity of the macropyhte *Stratiodes* *aloides* . J Appl Sci 10: 1769-1774. doi:10.3923/jas.2010.1769.1774.

[28] Hilt S, Ghobrial M, Gross EM (2006) *In* *situ* allelopathic potential of *Myriophyllum* *verticillatum* (Haloragaceae) against selected phytoplankton species. J Phycol 42: 1189-1198. doi:10.1111/j.1529-8817.2006.00286.x.

[29] Bauer N, Blaschke U, Beutler E, Gross EM, Jenett-Siems K et al. (2009) Seasonal and interannual dynamics of polyphenols in *Myriophyllum* *verticillatum* L. and its allelopathic activity on phytoplankton. Aquat Bot 91: 110-116. doi:10.1016/j.aquabot.2009.03.005.

[30] Komárek J, Fott B (1983) Das Phytoplankton des Süβwassers 7. Teil, 1 Hälfte. E. Schweizerbart’sche Verlasgbuchhandlung, Stuttgart, Germany.

[31] Vanormelingen P, Hegewald E, Braband A, Kitschke M, Friedl T et al. (2007) The systematics of a small spineless *Desmodesmus* taxon, *D*. *costato-granulatus* (sphaeropleales, Chlorophyceae), based on ITS2 rDNA sequence analyses and cell wall morphology. J Phycol 43: 378-396. doi:10.1111/j.1529-8817.2007.00325.x.

[32] Guillard RRL, Lorenzen CJ (1972) Yellow-green algae with chlorophyllide *c* . J Phycol 8: 10-14. doi:10.1111/j.0022-3646.1972.00010.x.

[33] Körner S, Nicklisch A (2002) Allelopathic growth inhibition of selected phytoplankton species by submerged macrophytes. J Phycol 38: 862-871. doi:10.1046/j.1529-8817.2002.t01-1-02001.x.

[34] Nicklisch A (1992) The interaction of irradiance and temperature on the growth rate of *Limnothrix* *redekei* and its mathematical description. Arch Hydrobiol Algol Stud 63 [Suppl.]: 1-18.

[35] McManus HA, Lewis LA (2011) Molecular phylogenetic relationships in the freshwater family Hydrodictyaceae (Sphaeropleales, Hydrodictyaceae), with an emphasis on *Pediastrum* *duplex* . J Phycol 47: 152-163. doi:10.1111/j.1529-8817.2010.00940.x. 27021721

[36] Buchheim M, Buchheim J, Carlson T, Braband A, Hepperle D et al. (2005) Phylogeny of the Hydrodictyaceae (Chlorophyceae): inferences from rDNA data. J Phycol 41: 1039-1054. doi:10.1111/j.1529-8817.2005.00129.x.

[37] Zechman FW, Zimmer EA, Theriot EC (1994) Use of ribosomal DNA internal transcribed spacers for phylogenetic studies in diatoms. J Phycol 30: 507-512. doi:10.1111/j.0022-3646.1994.00507.x.

[38] Ronquist F, Huelsenbeck JP (2003) MRBAYES 3: Bayesian phylogenetic inference undermixed models. Bioinformatics 19: 1572–1574. doi:10.1093/bioinformatics/btg180. PubMed: 12912839. 12912839

[39] Schreiber U (1996) Detection of rapid induction kinetics with a new type of high- frequency modulated chlorophyll fluorometer. Photosynth Res 9: 261-272. 10.1007/BF00029749 24442302

[40] Lürling M, Verschoor AM (2003) F_0_-spectra of chlorophyll fluorescence for the determination of zooplankton grazing. Hydrobiologia 491: 145-157. doi:10.1023/A:1024436508387.

[41] Ting CS, Owens TG (1992) Limitations of the pulse-modulated technique for measuring the fluorescence characteristics of algae. Plant Physiol 100: 367-373. doi:10.1104/pp.100.1.367. PubMed: 16652970. 16652970 PMC1075560

[42] Juneau P, Harrison PJ (2005) Comparison by PAM fluorometry of photosynthetic activity of nine marine phytoplankton grown under identical conditions. Photochem Photobiol 81: 649-653. doi:10.1562/2005-01-13-RA-414.1. PubMed: 15686444. 15686444

[43] Planas D, Sarhan F, Dube L, Godmaire H (1981) Ecological significance of phenolic compounds of *Myriophyllum* *spicatum* . Verh Internat Verein Limnol 21: 1492-1496.

[44] Eigemann F, Hilt S, Schmitt-Jansen M (2013) Flow cytometry as a diagnostic tool for the effects of polyphenolic allelochemicals on phytoplankton. Aquat Bot 104: 5-14. doi:10.1016/j.aquabot.2012.10.005.

[45] Hilt S, Beutler E, Bauer N (2012) Comparison of methods to detect allelopathic effects of submerged macrophytes on green algae. J Phycol 48: 40-44. doi:10.1111/j.1529-8817.2011.01106.x. 27009648

[46] Reusch TBH, Ehlers A, Hämmerli A, Worm B (2005) Ecosystem recovery after climatic extremes enhanced by genotypic diversity. Proc Natl Acad Sci USA 102: 2826-2831. doi:10.1073/pnas.0500008102. PubMed: 15710890. 15710890 PMC549506

[47] Carillo E, Ferrero LM, Alonso-Andicoberry C, Basanta A, Martin A et al. (2003) Interstrain variability in toxin production in populations of the cyanobacterium *Microcystis* *aeruginosa* from water-supply reservoirs of Andalusia and lagoons of Donana National Park (southern Spain). Phycologia 42: 269–274. doi:10.2216/i0031-8884-42-3-269.1.

[48] Inderjit, Wardle DA, Karban R, Callaway RM (2011) The ecosystem and evolutionary context of allelopathy. Trends Ecol Evol 1442: 1-8. 10.1016/j.tree.2011.08.003 21920626

[49] Leu E, Krieger-Liszkay A, Goussias C, Gross EM (2002) Polyphenolic allelochemicals from the aquatic angiosperm *Myriophyllum* *spicatum* inhibit photosystem II. Plant Physiol 130: 2011-2018. doi:10.1104/pp.011593. PubMed: 12481084. 12481084 PMC166712

[50] Mulderij G, Mooij WM, Smolders AJP, Van Donk E (2005) Allelopathic inhibition of phytoplankton by exudates from *Stratiodes* *aloides* . Aquat Bot 82: 284-296. doi:10.1016/j.aquabot.2005.04.001.

[51] Falkowsky PG (1984) Kinetics of adaptation to irradiance in *Dunaliella* *tertiolecta* . Photosynthetica 18: 62-66.

[52] Genty B, Briantais J, Naker NR (1989) The relationship between the quantum yield of photosynthetic electron transport and quenching of chlorophyll fluorescence. Biochim Biophys Acta 990: 87-92. doi:10.1016/S0304-4165(89)80016-9.

[53] Bonaventura C, Myers J (1969) Fluorescence and oxygen evolution from *Chlorella* *pyrenoidosa* . Biochim Biophys Acta 189: 366-383. doi:10.1016/0005-2728(69)90168-6. PubMed: 5370012. 5370012

[54] Campbell D, Hurry V, Clarke AK, Gustafsson P, Öqusit G (1998) Chlorophyll fluorescence analysis of cyanobacterial photosynthesis and acclimation. Microbiol Mol Biol Rev 62: 667-683. PubMed: 9729605. 9729605 10.1128/mmbr.62.3.667-683.1998 PMC98930

[55] Spijkerman E (2010) High photosynthetic rates under a colimitation for inorganic phosphorus and carbon dioxide. J Phycol 46: 658-664. doi:10.1111/j.1529-8817.2010.00859.x.

[56] Bährs H, Steinberg CEW (2012) Impact of two different humic substances on selected coccal green algae and cyanobacteria – changes in growth and photosynthetic performance. Environ Sci Pollut Res 19: 335-346. doi:10.1007/s11356-011-0564-7. 21751018

[57] Low-Décarie E, Jewell MD, Fussmann GF, Bell G (2013) Long-term culture at elevated atmospheric CO_2_ fails to evoke specific adaptation in seven freshwater phytoplankton species. Proc R Soc Lond B 280: 20122598 PubMed: 23303540. 10.1098/rspb.2012.2598 PMC3574323 23303540

[58] de Meester L (1996) Local genetic differentiation and adaptation in freshwater zooplankton populations: patterns and processes. EcoScience 3: 385-399.

[59] Rynearson TA, Armbrust EV (2005) Maintenance of clonal diversity during a spring bloom of the centric diatom *Ditylum* *brightwellii* . Mol Ecol 14: 1631-1640. doi:10.1111/j.1365-294X.2005.02526.x. PubMed: 15836638. 15836638

[60] Reed DH, Lowe EH, Briscoe DA, Frankham R (2003) Fitness and adaptation in a novel environment: effect of inbreeding, prior environment, and lineage. Evolution 57: 1822-1828. doi:10.1554/02-601. PubMed: 14503623. 14503623

